# Research on ADR1s helps understanding the plant immune network

**DOI:** 10.1007/s44154-022-00038-1

**Published:** 2022-02-07

**Authors:** Meijuan Hu, Jian-Min Zhou

**Affiliations:** 1 State Key Laboratory of Plant Genomics, Institute of Genetics and Developmental Biology, Innovation Academy for Seed Design, Chinese Academy of Sciences, Beijing, 100101 China; 2 CAS Center for Excellence in Biotic Interactions, University of Chinese Academy of Sciences, Beijing, 100049 China

**Keywords:** Immune receptors, PTI, ETI, Resistance, ADR1

## Abstract

Plant innate immunity begins with the recognition of pathogens by plasma membrane localized pattern-recognition receptors (PRRs) and intracellular nucleotide-binding domain leucine-rich repeat containing receptors (NLRs), which lead to pattern-triggered immunity (PTI) and effector-triggered immunity (ETI), respectively. For a long time, PTI and ETI have been regarded as two independent processes although they share multiple components and signal outputs. Increasing evidence shows an intimate link between PTI and ETI. PTI and ETI mutually potentiate each other, and this is essential for robust disease resistance during pathogen infection. An ancient class of NLRs called RNLs, so named because they carry a Resistance to Powdery Mildew 8 (RPW8)-like coiled-coil (CC) domain in the N terminus, has emerged as a key node connecting PTI and ETI. RNLs not only act as helper NLRs that signal downstream of sensor NLRs, they also directly mediate PTI signaling by associating with PRR complexes. Here, we focus on Activated Disease Resistance 1 (ADR1), a subclass of RNLs, and discuss its role and mechanism in plant immunity.

## Main text

Plants utilize cell surface-localized pattern-recognition receptors (PRRs) and intracellular nucleotide-binding domain leucine-rich repeat containing receptors (NLRs) to perceive pathogens and activate immunity. PRRs, comprising receptor kinases (RKs) and receptor-like proteins (RLPs), detect microbe- or plant-derived immunogenic molecular patterns from outside of the plant cell to cause pattern-triggered immunity (PTI). NLRs recognize pathogen effectors delivered inside the plant cell that are originally evolved as virulence factors, leading to effector-triggered immunity (ETI). PTI and ETI share a number of common signaling outputs, including reactive oxygen species (ROS) burst, calcium ion (Ca^2+^) influx, mitogen-activated protein kinases (MAPKs) activation, transcriptional reprogramming and biosynthesis of defense hormones such as salicylic acid (SA) and ethylene. ETI is more robust than PTI and often causes hypersensitive response (HR), a type of programmed cell death that limits pathogen proliferation at the infection site (Wang et al., 2020).

Plant NLRs can be classified into three major classes based on their N terminal domain. Thus NLRs carrying Toll/interleukin-1 receptor (TIR) domain, coiled-coil (CC) domain, Resistance to Powdery Mildew 8 (RPW8)-like CC domain are referred to as TNLs, CNLs, and RNLs, respectively. Many TNLs and CNLs directly or indirectly recognize pathogen effectors, thus are regarded as sensor NLRs. In contrast, RNLs do not play a role in sensing effectors. Instead, they transduce immune signals from sensor NLRs to activate cell death and immune responses, thus are regarded as helper NLRs (Jubic et al., 2019).

Contrary to the thinking that PTI and ETI operate independently, two recent studies elegantly showed that PTI and ETI mutually potentiate each other (Ngou et al., 2021; Yuan et al., 2021). Although the activation of ETI alone is sufficient to induce transcription of numerous genes encoding PTI components, it induces only weak ROS and HR and is insufficient for disease resistance to incompatible bacteria. Co-activation of both PTI and ETI leads to much stronger defenses than those activated by PTI or ETI alone (Ngou et al., 2021; Yuan et al., 2021). However, how PTI and ETI potentiate each other remains largely unknown.

In recent years, major breakthroughs have been made not only on mechanisms of NLR activation, but also signaling. The Arabidopsis sensor CNL ZAR1 undergoes dramatic conformational changes upon activation and oligomerizes to form a pentameric complex, termed resistosome (Wang et al., 2019a; Wang et al., 2019b). This brings the CC domain into the center of the protein complex and forms a Ca^2+^-permeable cation channel at plasma membrane (PM), leading to Ca^2+^ influx that initiate downstream signaling which culminate in cell death and immunity (Bi et al., 2021). Two TNLs, RPP1 and Roq1, form tetrameric resistosomes upon recognition of effectors (Ma et al., 2020; Martin et al., 2020). The TIR domains of TNLs are NADases, which can cleave NAD^+^ to produce small signaling molecules (Horsefield et al., 2019; Wan et al., 2019). The clustering of the TIR domains in the tetrameric resistosome is responsible for the activation of the NADase activity.

**Fig. 1:**
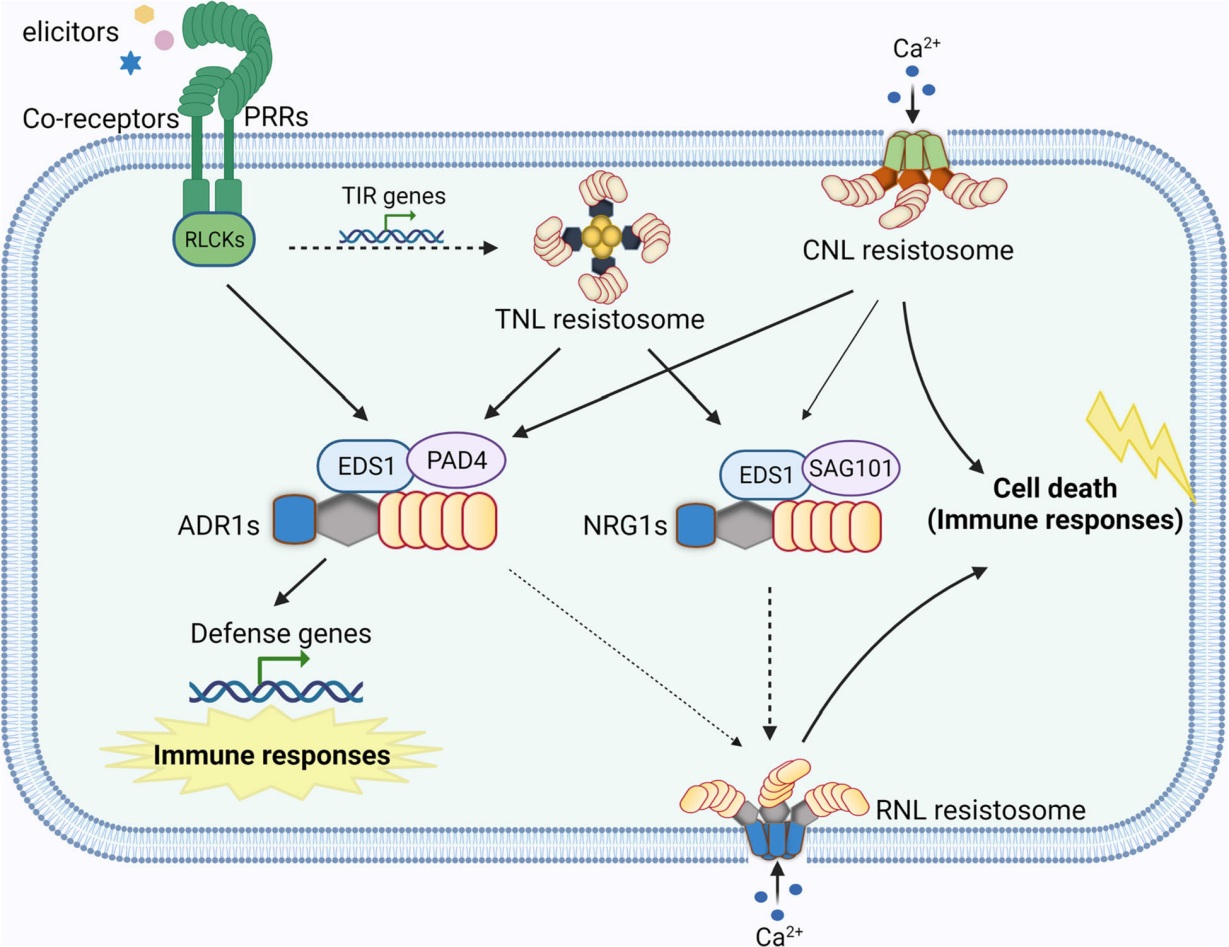
Proposed model of RNL function in plant immune network. Elicitor perception by PRRs activate downstream signaling mainly via receptor-like cytoplasmic kinases (RLCKs). CNL resistosomes function as Ca^2+^-permeable channels at PM. RNL members ADR1s and NRG1s function together with EDS1-PAD4 and EDS1-SAG101, respectively, to transduce signals from PRRs and NLRs. The activation of PRRs may induce the expression of TIR genes, leading to activation of immune responses through TNL pathways. The EDS1-PAD4-ADR1 module associates with PRR complexes, which may directly mediate PTI signaling. The EDS1-PAD4-ADR1 and EDS1-SAG101-NRG1 modules also function downstream of sensor NLRs (TNLs and CNLs). The EDS1-PAD4-ADR1 module is necessary for transcriptional activation of defense-related genes, limiting the growth of pathogens, and plays a minor role in inducing cell death. The EDS1-SAG101-NRG1 module is indispensable for cell death triggered by all TNLs tested to date. Upon activation, ADR1s and NRG1s may oligomerize to form RNL resistosomes, which function as Ca^2+^-permeable channels at PM, leading to calcium influx and eventual cell death and immunity. Solid lines indicate processes supported by literatures, whereas dashed lines represent hypothetical processes. Thin arrows (from CNL resistosome to NRG1s, and ADR1s to RNL resistosome) suggest minor contributions

RNLs are further divided into two subfamilies, the Activated Disease Resistance 1 (ADR1) subfamily, and the N Required Gene 1 (NRG1) subfamily. Most monocots only carry ADR1 subfamily and have lost the NRG1 subfamily during evolution. The Arabidopsis ADR1 subfamily contains four members, including three full-length ADR1, ADR1-L1 and ADR1-L2 and an N-terminal truncated ADR1-L3, while the NRG1 subfamily contains two full-length members NRG1.1 and NRG1.2 and an N-terminal truncated NRG1.3 (Jubic et al., 2019). The Arabidopsis ADR1s and NRG1s are involved in ETI through unequal redundant functions. ADR1s are required for disease resistance mediated by TNLs and some CNLs. For example, full resistance to incompatible pathogens conferred by TNLs RPP2, RPP4 and RPS4 and CNLs RPS2 and RPS5 depends on ADR1s (Saile et al., 2020). ADR1s function together with a heterodimer composed of lipase-like proteins Enhanced Disease Susceptibility 1 (EDS1) and Phytoalexin Deficient 4 (PAD4) and play an important role in increasing the biosynthesis of SA and activation of SA-related genes (Bonardi et al., 2011; Jubic et al., 2019; Wang et al., 2020). Although ADR1s do not contribute to the disease resistance mediated by CNLs RPM1 and ZAR1, optimum induction of SA-related genes by these CNLs is still dependent on ADR1s, indicating a broad role of ADR1s in immune signaling downstream of both TNLs and CNLs (Saile et al., 2020). NRG1s function exclusively with EDS1 and another lipase-like protein Senescence-Associated Gene101 (SAG101), and are primarily required for cell death downstream of all TNLs tested to date (Jubic et al., 2019; Saile et al., 2020; Sun et al., 2021). Activation of TNLs lead to recruitment of NRG1 to the EDS1-SAG101 complex (Sun et al., 2021). Thus, EDS1-PAD4-ADR1 and EDS1-SAG101-NRG1 are regarded as two important signal transduction modules downstream of sensor NLRs, particularly in TNL signaling (Fig. 1).

In addition to their indispensable role in ETI, RNLs are also necessary for PTI activation. Recent advances uncovered remarkable crosstalk between PRR- and NLR-mediated signaling connected by ADR1 members. Independent studies by Pruitt et al. and Tian et al. convincingly showed that ETI signaling components EDS1, PAD4 and ADR1s are required for PTI activation. Pruitt et al. found that the EDS1-PAD4-ADR1 module is essential for PTI signaling, particularly in RLP-initiated signaling (Pruitt et al., 2021). In Arabidopsis, RLP-dependent induction of ethylene production, ROS burst, callose deposition and resistance against *Pseudomonas syringae* pv. *tomato* (*Pst*) are greatly diminished in mutants defective in the EDS1-PAD4-ADR1 module, demonstrating a shared requirement of this module for PTI and ETI signaling (Pruitt et al., 2021). In addition, RLP23 and co-receptor SOBIR1 are spatially close to EDS1-PAD4-ADR1, suggesting that they form a constitutive complex at PM and that EDS1-PAD4-ADR1 likely function immediately downstream of RLP23-SOBIR1 (Pruitt et al., 2021). The parallel study by Tian et al. showed that RK- and RLP-mediated defense genes expression, SA biosynthesis and resistance to virulent pathogens are primarily dependent on EDS1, PAD4 and ADR1s and modestly dependent on NRG1s and SAG101 in Arabidopsis (Tian et al., 2021). Furthermore, activation of PTI can upregulate the expression of a number of *TNLs*, and overexpression of some of these *TNLs* in *Nicotiana benthamiana* and Arabidopsis led to increased defense outputs, suggesting induction of *TNLs* by PTI can boost immune responses through TNL signaling pathways (Tian et al., 2021). These findings indicate that EDS1-PAD4-ADR1 is an important node linking PTI and ETI in Arabidopsis (Fig. 1).

Similar to ZAR1, recent studies showed that the auto-active variants of NRG1.1 and ADR1 oligomerize to form Ca^2+^-permeable channels at PM, and these are indispensable for the induction of Ca^2+^ influx and cell death (Jacob et al., 2021). Also, Saile et al. showed that ADR1s directly interact with negatively charged phospholipids of the PM and form homo- and heteromeric complexes (Saile et al., 2021). Further, Wu et al. found that TIR signal can trigger the interaction between ADR1-L1 (Wu et al., 2021). These findings support the possibility that RNLs form oligomeric complexes upon activation and function as Ca^2+^-permeable channels at PM. Because RNLs are indispensable for TNL signaling, it can be inferred that TNLs also initiate immune signaling through calcium flux. Thus calcium flux appears to be the initial cellular signal for immune activation by both CNLs and TNLs.

Do ADR1s have any other functions besides Ca^2+^ channels? Previous studies found that the SA biosynthesis and defenses activated by RPS2 require ADR1s, but the intact P-loop of ADR1-L2 is dispensable for these processes (Bonardi et al., 2011). As P-loop is required for oligomerization, suggesting ADR1s may have other functions beyond oligomerization and formation of Ca^2+^ channels. Saile et al. showed that ADR1 P-loop mutant proteins are located not only at PM but also in the cytosol and endoplasmic reticulum, suggesting that ADR1s may regulate SA biosynthesis and defenses through a mechanism independent of the ADR1 channel function (Saile et al., 2021). Recently, Wu et al. showed that ADR1-L1 directly interacts with EDS1-PAD4, which is enhanced by TIR signal (Wu et al., 2021). EDS1 is located in both the cytosol and nucleus, and an increase of EDS1 nuclear accumulation precedes EDS1-dependent transcriptional reprogramming (Garcia et al., 2010). Thus, it will be interesting to test whether activation of any sensor NLRs can trigger nuclear translocation of ADR1s for transcriptional reprogramming. If yes, whether it involves oligomerization.

In summary, exciting breakthroughs have been made on the mode of action of NLRs and crosstalk between NLR- and PRR-mediated signaling. These studies with previous findings provide exciting insights of how ADR1s integrate ETI and PTI signal transduction (Fig. 1). However, several open questions remain. Does the EDS1-PAD4-ADR1 module regulate PTI and ETI via similar or different mechanisms? Although ADR1s can form ion channels, it remains unknown whether the activation of PRRs triggers oligomerization of ADR1s. Second, it is not clear whether ADR1s have other functions beyond forming Ca^2+^ channels at PM. Previous studies have clearly shown a nuclear function of EDS1 in immune activation (Garcia et al., 2010). It remains unknown whether and how this function is linked to ADR1s. Transcriptional activation of PTI components is key to the ETI-PTI crosstalk. It is not known, however, whether and how the Ca^2+^ influx triggered by CNLs and RNLs induce the expression of these PTI components. Answering these questions will further advance our understanding of the plant immune network.

## Data Availability

Not applicable.

## Abbreviations

ADR1: Activated Disease Resistance 1
CC: Coiled-coil
CNLs: CC NLRs
EDS1: Enhanced Disease Susceptibility 1
ETI: Effector-triggered immunity
HR: Hypersensitive response
MAPKs: Mitogen-activated protein kinases
NLRs: Nucleotide-binding domain leucine-rich repeat containing receptors
NRG1: N Required Gene 1
PAD4: Phytoalexin Deficient 4
PM: Plasma membrane
PRRs: Pattern-recognition receptors
*Pst*: *Pseudomonas syringae* pv*. tomato*
PTI: Pattern-triggered immunity
RKs: Receptor kinases
RLCKs: Receptor-like cytoplasmic kinases
RLPs: Receptor-like proteins
RNLs: RPW8-like CC NLRs
ROS: Reactive oxygen species
RPW8: Resistance to Powdery Mildew 8
SA: Salicylic acid
SAG101: Senescence-Associated Gene101
TIR: Toll/interleukin-1 receptor
TNLs: TIR NLRs
